# Calendar-defined missed-workday gaps in radiotherapy: a site- and phase-stratified audit with linkage sensitivity analyses

**DOI:** 10.3389/fonc.2026.1932765

**Published:** 2026-10-07

**Authors:** Runhong Lei, Yongping Zheng, Yi Chen, Haitao Sun, Ang Qu, Ping Jiang

**Affiliations:** Department of Radiation Oncology, Peking University Third Hospital, Beijing, China

**Keywords:** calendar-defined missed workday, MOSAIQ, operational audit, overall treatment time, radiotherapy quality assurance, treatment continuity

## Abstract

**Background:**

Missed treatment workdays can prolong overall treatment time and may reduce tumor control, yet routine audits often conflate weekends, statutory holidays, and departmentally accepted rest schedules with untreated institutional workdays.

**Methods:**

We quantified calendar-defined missed-workday gaps—inter-fraction intervals containing ≥1 untreated institutional workday after excluding routine rest days—among patients with first treatment between June 2021 and December 2025 who were linkable between the MOSAIQ radiotherapy information system and the institutional oncology database (1,319 patients; 1,342 episodes). A locked stratified sample of 100 algorithm-positive gaps underwent documentation-based chart review as unplanned relevant (UR) or planned/appropriate (PA). We analyzed linkage sensitivity, opportunity-adjusted site rates with patient-clustered confidence intervals (CIs), opportunity-offset Poisson generalized estimating equations (GEE) including calendar year, and episode-clustered phase GEE.

**Results:**

Under the documentation-based chart-review rule, 92 of 100 algorithm-positive gaps were labeled UR (17 department/equipment; 75 patient-factor) and 8 PA. Gaps occurred in 638/1,342 episodes (47.5%; patient-level 48.0%). Linked versus unlinked prevalence was 48.2% versus 51.7% (absolute difference −3.4 percentage points, 95% CI −11.2 to 4.4; P = 0.435). Among subgroups with ≥50 episodes, head and neck had the highest prevalence (75.3%) and breast the lowest (41.3%); opportunity-adjusted rates were 5.63 (cluster-bootstrap 95% CI 4.76–6.52) versus 2.63 (2.36–2.92) per 100 opportunities. Opportunity-offset Poisson GEE with calendar year gave a head-and-neck versus breast incidence rate ratio (IRR) of 2.99 (95% CI 2.18–4.11). An exploratory phase GEE (29,763 intervals; 1,323 clusters) showed a late versus early odds ratio (OR) of 1.31 (95% CI 1.12–1.54; P = 0.0009; absolute rates 2.8% vs 3.6%); after site, year, and intent adjustment, the OR was 1.32 (95% CI 1.12–1.57).

**Conclusions:**

This operational audit of institutional workdays without recorded treatment—rather than clinically confirmed unplanned interruptions—shows frequent gaps after excluding routine rest. Most reviewed positives received patient-factor labels under a documentation rule. Calendar-aware auditing may support departmental monitoring; priority-based compensation remains a proposed application.

## Highlights

- Calendar-aware MOSAIQ auditing that excluded weekends and statutory holidays identified missed-workday gaps in 47.5% of episodes (patient-level 48.0%).
- Chart review labeled 92/100 algorithm-positive gaps as unplanned relevant under a documentation-based rule (not a clinical positive predictive value); among those, patient-factor labels outnumbered documented department/equipment labels (75 vs 17).
- Head-and-neck sites had the highest episode-level rate (75.3%) and higher opportunity-adjusted gap density than breast (5.63 [4.76–6.52] vs 2.63 [2.36–2.92] per 100 opportunities).
- In an exploratory phase generalized estimating equation (GEE) analysis excluding short courses (≤8 treatment days), late-course gaps had higher odds than early-course gaps (odds ratio [OR] 1.31; 95% confidence interval [CI] 1.12–1.54; P = 0.0009), with absolute rates of 2.8% versus 3.6%.
- Linked and unlinked patients had gap prevalences of 48.2% versus 51.7% (absolute difference −3.4 percentage points, 95% CI −11.2 to 4.4).

## Introduction

The timely delivery of radical radiotherapy is an established quality indicator in radiation oncology and a prerequisite for optimal tumor control. Fractionated radiotherapy exploits differences in repair capacity between malignant and normal tissues; however, when treatment is prolonged, surviving clonogenic tumor cells may proliferate during inter-fraction intervals, partially offsetting the biological effect of delivered dose (1). Classic radiobiologic modeling demonstrated that accelerated repopulation can become clinically relevant after approximately four to five weeks of treatment, particularly in rapidly proliferating epithelial malignancies (1, 2). Empirical studies in cervical, head-and-neck, and lung cancers have linked prolongation of overall treatment time (OTT) to inferior local control, with each additional week of delay associated with measurable decrements in outcome in selected disease sites (2–4).

International guidance has therefore emphasized minimizing unscheduled treatment gaps and implementing compensation when interruptions occur. The Royal College of Radiologists (RCR) fourth-edition guidelines categorize tumors by repopulation potential and recommend escalation pathways for high-priority histologies when unplanned gaps exceed defined thresholds (5). Similar principles have been reiterated in clinical oncology reviews advocating prospective interruption registries, early identification of at-risk patients, and standardized compensation algorithms (6, 7). Despite this consensus, implementation remains heterogeneous across institutions, and many departments lack electronic tools that distinguish scheduled rest days from missed workdays that may need review (8, 9).

Retrospective audits consistently demonstrate that treatment interruptions are common in real-world practice. A multicenter European rectal cancer audit reported treatment interruptions of at least one day in 43.9% of patients, with wide inter-center variability (2.5%–90%) and compensation performed in only 2.1% of cases (9). Spanish and Italian experience similarly found that most patients experienced at least one day of OTT extension, with machine downtime, acute toxicity, and patient noncompliance among leading causes (8, 10). Head-and-neck series have documented interruption prevalence exceeding 70% in some settings, supporting site-specific surveillance (11). Predictive models incorporating sociodemographic and treatment variables have been proposed to identify patients at risk of noncompletion, although external validation remains limited (12, 13).

Not all calendar gaps between fractions represent equivalent quality failures. Many departments routinely suspend treatment on weekends and statutory holidays; during extended national holidays, centers may either accept prolonged calendar gaps or deliberately treat on rest days to preserve biological effect. Conventional metrics that count all non-treatment days as interruptions therefore overestimate clinically relevant delays and may inappropriately penalize departments following accepted scheduling conventions (5, 7). Compensation models likewise assume knowledge of which missed days require dose recovery (7, 14). Without calendar-aware definitions, quality audits may count scheduled rest intervals together with missed workdays.

Scheduling context is particularly important in mainland China, where Saturday–Sunday weekends, national statutory holidays, and occasional adjusted workdays create a distinctive treatment calendar. Extended holidays such as the Lunar New Year and National Day Golden Week may produce multi-day calendar gaps that are operationally planned rather than clinically unplanned. Prior interruption studies from Asia have documented prolonged OTT in breast and nasopharyngeal cohorts but have rarely integrated automated statutory-holiday classification with departmental rest policies (15, 16). Complementary holiday-focused analyses likewise show that national-holiday interruptions can affect postoperative head-and-neck radiotherapy and nasopharyngeal carcinoma outcomes (17, 18). The operational burden of missed workdays in contemporary Chinese radiotherapy practice therefore remains incompletely described.

The study interval (June 2021–December 2025) partially overlapped national COVID-19 control periods. At our institution, radiotherapy patients were subject to special continuity-oriented policies; although a minority experienced brief routine-treatment gaps of a few days, pandemic-related disruption was not quantified as a separate analytic stratum.

Interruption risk may not be uniform across the treatment course. Late-course gaps may reflect cumulative acute toxicity, transportation fatigue, or equipment constraints, whereas early interruptions may signal access barriers or comorbidity-related deferrals (11, 19). Site-specific differences are also expected: rapidly proliferating head-and-neck malignancies are considered highly sensitive to OTT prolongation (4, 11, 20), whereas selected breast and prostate schedules may tolerate brief delays, although prolonged gaps remain undesirable (15). Anatomic site, treatment duration, and tumor priority should therefore be examined jointly rather than reporting a single center-wide interruption rate.

Electronic record systems such as the MOSAIQ radiotherapy information system capture daily treatment delivery with timestamp precision, enabling reconstruction of inter-fraction intervals at scale. Linkage to institutional diagnosis and treatment-site registries further permits stratification by disease category and anatomic site. Such linkages support departmental quality audits, particularly when reporting follows the Strengthening the Reporting of Observational Studies in Epidemiology (STROBE) recommendations for observational research (21).

This retrospective cohort included consecutive patients with first treatment between June 2021 and December 2025 who were linkable between MOSAIQ treatment records and the institutional oncology database. Using a calendar-aware framework, we audited calendar-defined missed-workday gaps as an operational indicator of institutional workdays without recorded treatment (missing workdays after excluding weekends, statutory holidays, and scheduled long-holiday rest blocks), described early/middle/late course timing, compared treatment intensity and calendar span by gap status, and performed prespecified site-stratified analyses when stratum size met the reporting threshold. We hypothesized that gap rates would remain substantial after excluding routine rest days, would show an exploratory late-course pattern, and would be highest among head-and-neck patients.

## Materials and methods

### Study design and population

This retrospective observational study included patients who received external-beam radiotherapy at Peking University Third Hospital with first treatment between June, 2021 and December, 2025, captured on the MOSAIQ-linked linear accelerator under study. All analyzed fractions were delivered on this machine. Under a departmental rule intended to preserve dose-traceability, patients treated on this accelerator were not transferred to other machines within the same treatment course; episode reconstruction from the study-machine MOSAIQ log therefore represents the complete fraction sequence for that course. Eligible patients were required to (1) have a valid institutional radiation number and standard Chinese name in MOSAIQ; (2) be matchable to the institutional oncology database by radiation number and name, yielding clinical diagnosis and treatment site; and (3) have at least one recorded treatment fraction after data cleaning. The analytic machine log is breast-predominant by institutional allocation, with genitourinary and head-and-neck sites as the next main non-breast strata; case-mix implications are considered in the Discussion. The analyzable cohort comprised 1,319 unique patients contributing 1,342 treatment episodes; all eligible episodes within the study period were included in primary analyses. The study was approved by the Institutional Review Board of Peking University Third Hospital (Approval No. IRB00006761-M20260575); individual patient consent was waived for this retrospective quality audit.

Patient selection followed the flow summarized in **Figure 1**. After MOSAIQ record cleaning (344,008 beam records → 273,596 treatment fraction records), 4,285 unique patients remained. Of these, 1,322 were linkable to the institutional oncology database; 2,963 were not linkable. Among linked patients with first treatment between June 2021 and December 2025, 1,319 unique patients contributed 1,342 analyzable episodes after prespecified re-treatment course splitting (28 timelines were split by algorithm; after date-window filtering of episode start dates, this yielded a net +23 episodes in the analytic set: 21 patients contributed 2 episodes, 1 contributed 3, and 6 retained only 1 in-window episode).

**Figure 1:**
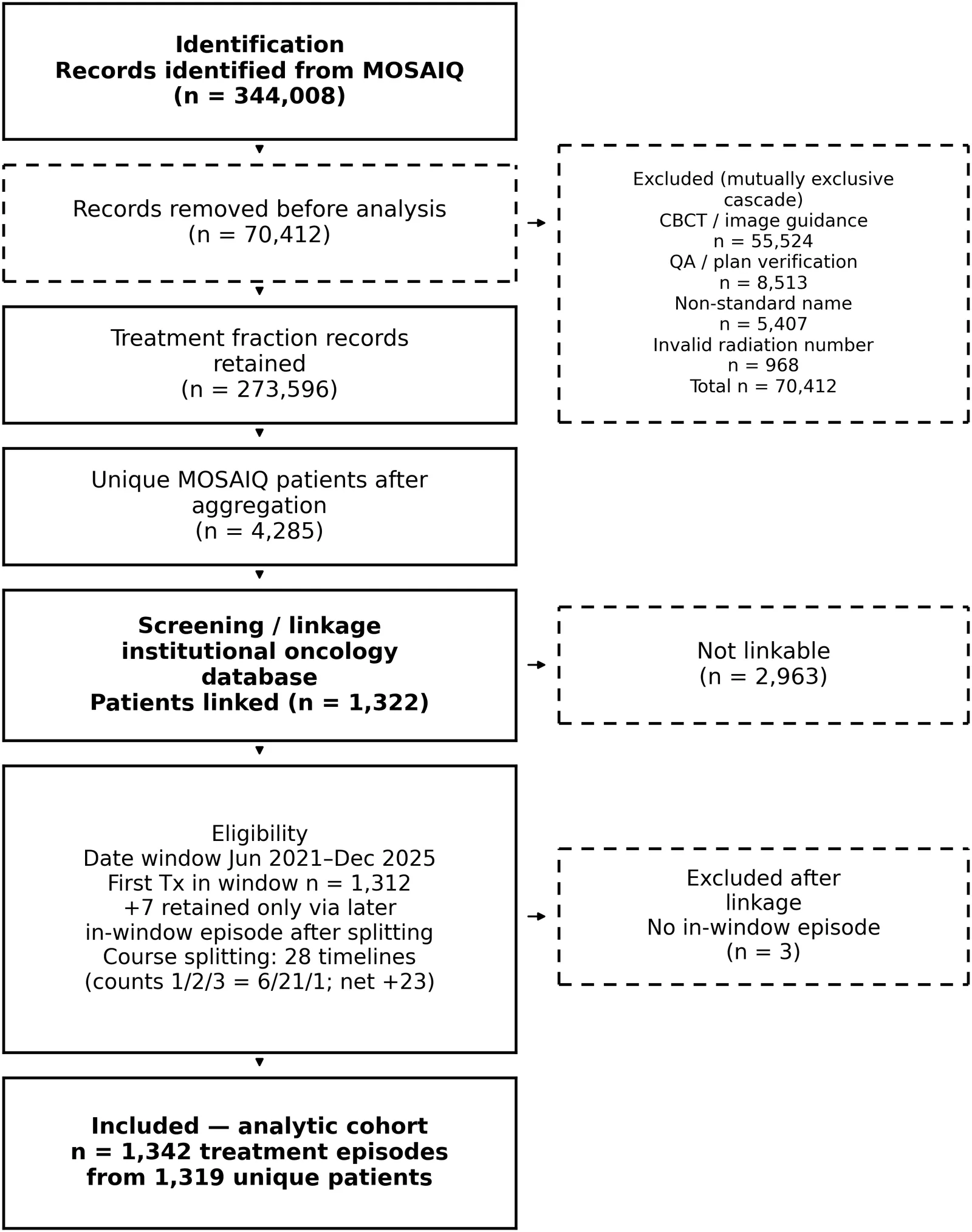
Participant and record flow from MOSAIQ identification through institutional-database linkage, date-window eligibility (June 2021–December 2025), and course splitting to the analytic cohort (1,342 treatment episodes from 1,319 unique patients). Exclusion reasons for nontherapeutic records are listed as a mutually exclusive cascade. Among linked patients, 1,312 had a first treatment in the study window; 7 were retained only via a later in-window episode after splitting; 3 had no in-window episode and were excluded. Course splitting flagged 28 timelines (in-window episode counts 1/2/3 = 6/21/1; net +23 episodes).

Linkage to the institutional oncology database was required for diagnosis, treatment site, and intent stratification. Earlier MOSAIQ delivery records were available, but contemporaneous institutional oncology data had not yet been upgraded into structured, scalable form; manual curation would have been prohibitively labor-intensive. Accordingly, the clinically linked analytic window began in June 2021. Even within this window, a minority of cleaned MOSAIQ patients remained unlinkable because of identifier mismatches or incomplete database coverage. We therefore compared delivery-record characteristics and calendar-defined gap prevalence between linked and unlinked patients in the same date window and estimated prevalence in the full cleaned MOSAIQ population as sensitivity analyses that do not require clinical-database fields.

### Data sources and preprocessing

Daily treatment data were extracted from MOSAIQ (Elekta). Records were excluded if the beam type indicated cone-beam computed tomography (CBCT)/image guidance (CT, KV Setup) or if the therapist field indicated quality-assurance or plan-verification sessions (QA), which do not represent patient treatment fractions. Non-standard patient identifiers and invalid radiation numbers were removed. For each patient-day, all remaining beam records were aggregated into a single treatment visit. Age recorded as 0 years in the institutional oncology database was treated as missing and excluded from age-specific descriptive statistics and age comparisons; affected episodes remained in all other analyses.

### Course episode splitting

Long treatment timelines were separated into independent episodes using date-clustering rules. Inter-fraction calendar gaps of ≥45 days defined course boundaries. Timelines with fraction counts exceeding disease-specific caps and/or calendar spans substantially longer than expected treatment duration were flagged for checking (caps: breast ≤33; prostate ≤40; other digestive/genitourinary ≤35; other external-beam ≤40; spans >60 days with substantial fraction counts were also reviewed). Twenty-eight such timelines underwent chart checking and algorithmic course splitting and yielded a net +23 in-window episodes after date filtering (size counts 1/2/3 = 6/21/1). Manual review agreed with the algorithm for 25/28 (89.3%). The algorithmic classification was retained for the three discordant timelines in the primary analysis. After excluding those three patients, episode-level gap prevalence remained 47.5% (44.8–50.1%; 634/1,336). After collapsing the one over-split timeline to a single chart-concordant episode (two under-split timelines retained algorithmic episodes because new boundaries were not reconstructable from the review sheet alone), prevalence was 47.6% (44.9–50.3%; 638/1,341). Alternative splitting thresholds left overall prevalence 47.2–47.9% Alternative splitting thresholds and discordant-case detail are reported with the **Supplementary Material**.

### Calendar-aware definitions of missed-workday gaps

For each patient, treatment dates were sorted chronologically. For every consecutive pair of treatment days, the open interval between dates was evaluated day-by-day using the Chinese mainland statutory holiday calendar (including adjusted workdays). Gaps were classified as follows: (i) no gap (adjacent calendar days); (ii) routine rest gap—every intervening day was a weekend or statutory holiday; (iii) long-holiday rest gap—entire gap fell within a >3-day consecutive rest block; (iv) calendar-defined missed-workday gap—at least one intervening workday without treatment. Calendar-defined missed-workday gaps were graded by missing workdays: mild (1–3), moderate (4–7), and severe (>7). Treatment delivered on a weekend or statutory holiday was defined as any treatment delivered on a weekend or statutory holiday, as shown in **Figure 2**.

**Figure 2:**
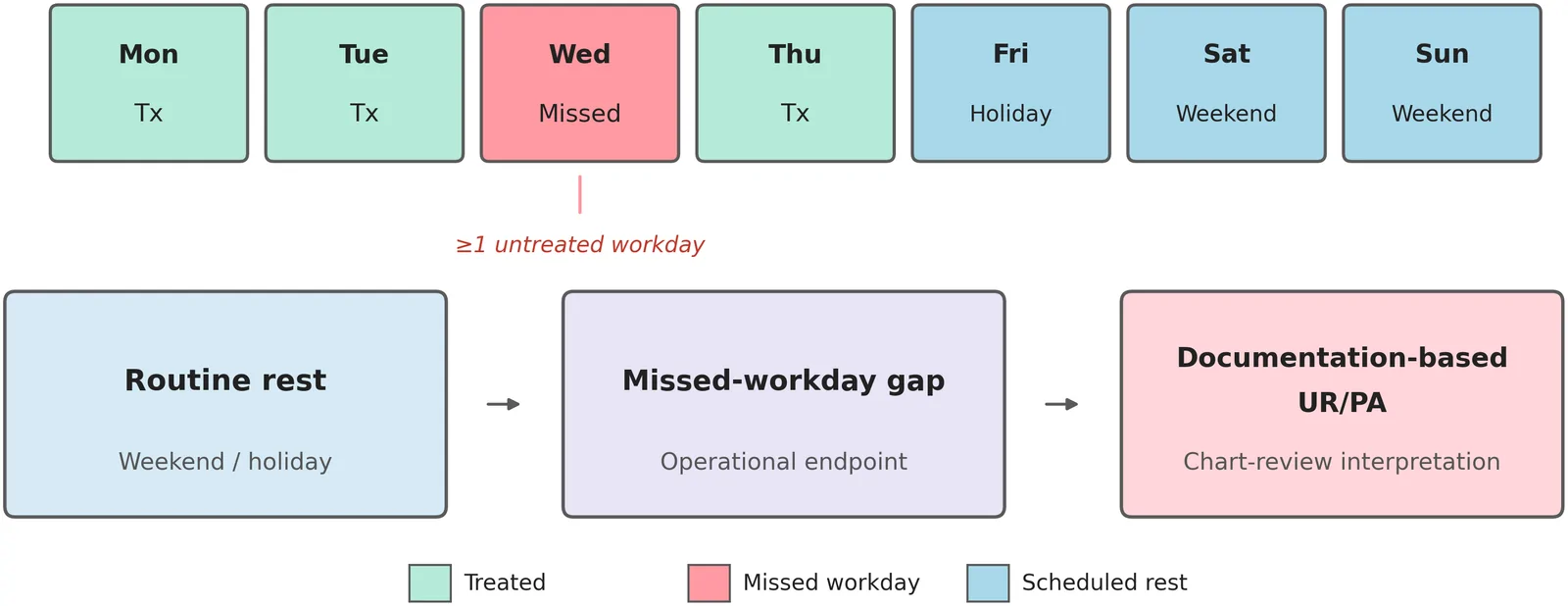
Schematic of calendar-aware gap classification. Top: illustrative Mon–Sun sequence with treated days (green), one missed workday (amber), and scheduled rest including a statutory holiday and weekend (blue). Bottom: routine rest is excluded from the operational endpoint; a calendar-defined missed-workday gap requires ≥1 untreated workday; chart-review labels (unplanned relevant vs planned/appropriate) are used for interpretation of selected algorithm-positive gaps and are not intended as a clinical gold-standard assessment of the full algorithm. Weekend/holiday treatment is reported descriptively and does not imply documented compensatory intent; planned long holidays may prolong overall treatment time and are analyzed separately from the operational gap endpoint. UR, unplanned relevant; PA, planned/appropriate.

### Chart review of algorithm-positive gaps

A locked stratified sample of 100 algorithm-positive gaps was reviewed using the locked worksheet of final classifications under the patient-factor documentation rule. Review was performed by the first author as a single reviewer. For each sampled gap, the reviewer inspected MOSAIQ delivery dates and institutional work-calendar classification, checked the departmental machine-fault/downtime calendar, and reviewed treatment scheduling adjacent to statutory holidays for planned holiday-adjacent adjustments. Strata were defined by treatment site (breast, head and neck, genitourinary, thorax, other), with a minimum head-and-neck quota (n=15); realized counts were breast 42, genitourinary 19, head and neck 15, other 13, and thorax 11. Sampling was not strictly proportional to the underlying positive-gap distribution; we therefore report the unweighted sample proportion under the documentation rule and do not claim a population-weighted clinical positive predictive value. Under institutional documentation practice, department-arranged missed treatments are recorded. Gaps with confirmed machine fault or department downtime were labeled unplanned relevant (UR; department/equipment); gaps with major-holiday adjacency and scheduling meant to preserve treatment continuity were labeled PA; remaining algorithm-positive gaps without a department stop-treatment note were labeled UR (patient-factor) by the documentation rule (rule-based labeling, not a chart-confirmed cause in every case). Of the 100 reviewed gaps, 92 were labeled UR (department/equipment 17; patient-factor 75) and 8 were labeled planned/appropriate (PA). The unweighted proportion labeled UR was 92.0% (85.0–95.9%). Cause labels from chart review provide contextual information on selected algorithm-positive gaps; they do not turn the operational endpoint into a clinically validated unplanned interruption. Algorithm-negative intervals were outside the scope of this chart review. An algorithm-negative interval indicates that MOSAIQ contains completed treatment records with documented fraction dose delivery on the relevant days; these completed records are strong evidence that fractions were delivered. Sensitivity and specificity against a separate chart gold standard for non-delivery were therefore not estimated.

### Course phase assignment

Each inter-fraction gap was assigned to early, middle, or late thirds of the completed fraction index. Short courses (≤8 treatment days; n=19 episodes) were retained in descriptive episode-level summaries but were excluded from the phase GEE analysis (29,763 inter-fraction intervals; 1,323 episode clusters). Disease-category and RCR-inspired priority mappings followed keyword rules in the **Supplementary Material**.

### Prespecified stratification and reporting thresholds

Stratified site and disease displays were prespecified for subgroups with ≥50 episodes to stabilize rate estimates. This threshold applied to stratified reporting only; all eligible episodes remained in primary cohort analyses.

### Computational pipeline and software

Analyses were run in Python with version-controlled scripts. Package versions and pipeline details are provided in **Supplementary Material**.

### Outcomes and statistical analysis

The primary endpoint was a calendar-defined missed-workday gap: an inter-fraction interval containing ≥1 untreated institutional workday after excluding weekends, statutory holidays, and scheduled long-holiday rest. This endpoint identifies workdays without recorded treatment on the institutional calendar. It does not, by itself, establish that the untreated day was included in an individual planned schedule, was clinically unplanned or inappropriate, or produced true overall-treatment-time prolongation relative to a prescribed expected completion date. Primary analyses reported episode-level prevalence of this operational gap and gap-level incidence by course phase. Secondary outcomes included maximum workday deficit, treatment intensity (treatment days ÷ calendar span), weekend/holiday treatment, and site-stratified rates (≥50-episode subgroups). Continuous variables are summarized as median (interquartile range [IQR]) and compared with two-sided Mann–Whitney U or Kruskal–Wallis tests as appropriate; categorical associations used chi-square tests. Two-sided P<0.05 defined statistical significance without multiplicity adjustment for these prespecified exploratory analyses. Interpretation emphasizes effect sizes, absolute differences, and confidence intervals rather than P values alone. Reporting followed STROBE guidance (21). Episode-level site rates used Wilson 95% confidence intervals (CIs). The treatment episode was the primary unit; patient-level prevalence and a one-episode-per-patient sensitivity analysis were also reported. Site comparisons distinguished ≥1-gap probability per episode from gaps per 100 inter-fraction opportunities; opportunity rates per 100 opportunities are reported with patient-clustered bootstrap 95% CIs. Confirmatory phase inference used episode-clustered binomial GEE (logit link; exchangeable working correlation; robust/sandwich standard errors), excluding episodes with ≤8 treatment days; odds ratios are reported with 95% CIs. An adjusted phase model additionally included site, calendar year, and treatment intent and is treated as exploratory. Primary multivariable site inference used patient-clustered Poisson GEE for gap counts with log(inter-fraction opportunities) as an offset, adjusting for age, sex, treatment intent, and calendar year of first treatment. Because the log offset is undefined when opportunities equal zero, episodes with fewer than two treatment days (n=6; all with zero inter-fraction intervals) were excluded, as were episodes with missing age (n=90; including two of the six single-day episodes). The resulting analytic sample was 1,248 episodes (1,230 patient clusters). A sensitivity analysis used patient-clustered binomial GEE with age, sex, treatment days, and intent; conventional logistic regression was also retained as sensitivity only. Calendar year of first treatment was included in the primary opportunity-offset site models; year-exclusion sensitivities and radical versus palliative summaries remained secondary. Longer calendar span and lower intensity among gap-positive episodes were descriptive coherence checks. Model diagnostics [variance inflation factor (VIF)/missingness] and unavailable confounders are summarized in the **Supplementary Material**.

## Results

### Patient characteristics

The analyzable cohort comprised 1,319 unique patients contributing 1,342 treatment episodes after re-treatment course splitting (28 timelines flagged; net +23 episodes). Breast sites accounted for 721/1,342 episodes (53.7%); genitourinary (n=224) and head-and-neck (n=93) were the main non-breast strata among ≥50-episode site groups (thorax n=179). Female patients accounted for 976 episodes (72.7%); male patients accounted for 366 episodes (27.3%). Median age was 56 years (IQR, 47–67 years; n=1,252). Age was missing for 90 episodes (recorded as 0 years in the source database). Overall, 638 episodes (47.5%) experienced at least one calendar-defined missed-workday gap (**Table 1**).

**Table 1:** Baseline characteristics of patients treated during the study period.

Variable	Value
Unique patients, n	1,319
Treatment episodes analyzed, n	1,342
Re-treatment courses split, n	28
Female episodes, n (%)	976 (72.7)
Male episodes, n (%)	366 (27.3)
Age, median (IQR), y	56 (47–67); n=1,252
Treatment days, median (IQR)	25 (23–25)
Calendar span, median (IQR), d	35 (32–37)
Treatment intensity, median (IQR)	0.694 (0.667–0.714)
Calendar-defined missed-workday gap, n (%)	638 (47.5)

Age excludes 90 episodes with missing age (0 years in source database).

### Gap burden and linkage sensitivity

Among 1,342 treatment episodes, 638 (47.5%) experienced at least one calendar-defined missed-workday gap. At the gap level, 948 of 29,823 inter-fraction intervals (3.2%) met criteria for calendar-defined missed-workday gap. The median missing workdays per calendar-defined missed-workday gap was 1 (mean 1.7). Severity distribution: no calendar-defined missed-workday gap 704; mild 559; moderate 54; severe 25. At the patient level, 633/1,319 patients (48.0%) experienced ≥1 gap in any episode; restricting to one episode per patient yielded 47.5% (44.9–50.2%).

### Linkage sensitivity

Among cleaned MOSAIQ patients with first treatment in the study window (n=1,492), 1,312 (87.9%) linked to the institutional database and 180 remained unlinked. Patient-level calendar-defined gap prevalence was 48.2% (45.6–51.0%) in linked patients and 51.7% (44.4–58.9%) in unlinked patients (absolute difference −3.4 percentage points, 95% CI −11.2 to 4.4; chi-square P = 0.435). Median treatment days were identical (25 vs 25), and median calendar span was 35 versus 36 days (Mann–Whitney P = 0.416). In the full cleaned MOSAIQ population irrespective of year (n=4,285), patient-level prevalence was 55.1% (53.7–56.6%); this denominator includes pre-2021 treatment eras outside the primary linkage window. Detailed linked versus unlinked comparisons are provided in **Supplementary eTable 1**.

### Chart review of algorithm-positive gaps

Among 100 locked algorithm-positive gaps (stratified by site with a head-and-neck minimum quota; unweighted), 92 (92.0% (85.0–95.9%)) were labeled unplanned relevant under the documentation-based chart-review rule and 8 as planned/appropriate holiday-adjacent arrangements. Of the 92 unplanned-relevant labels, 17 had documented department/equipment downtime and 75 were labeled patient-factor by a documentation-based rule when no department stop-treatment note existed. This 92/100 figure is the unweighted sample proportion under that documentation rule and is not a clinical positive predictive value. Patient-factor labels were applied when no department stop-treatment note existed.

### Calendar span and intensity by gap status

episodes with a calendar-defined missed-workday gap had longer calendar span (P<0.001) and lower treatment intensity (P<0.001) than episodes without calendar-defined missed-workday gap (**Table 2**; **Figure 3**). Treatment days were slightly higher in the gap-positive group (P<0.001), whereas age was significantly higher in the gap-positive group (median 58 vs 56 years; P = 0.044). Calendar span varied across prespecified anatomic sites (**Figure 4**).

**Table 2:** Comparison of episodes with versus without calendar-defined missed-workday gap (Mann-Whitney U test).

Variable	With gap (n=638)	Without gap (n=704)	P value
Treatment days, median	25	25	<0.001
Calendar span, median, d	36	34	<0.001
Treatment intensity, median	0.674	0.714	<0.001
Age, median, y	58 (n=588)	56 (n=664)	0.044

**Figure 3:**
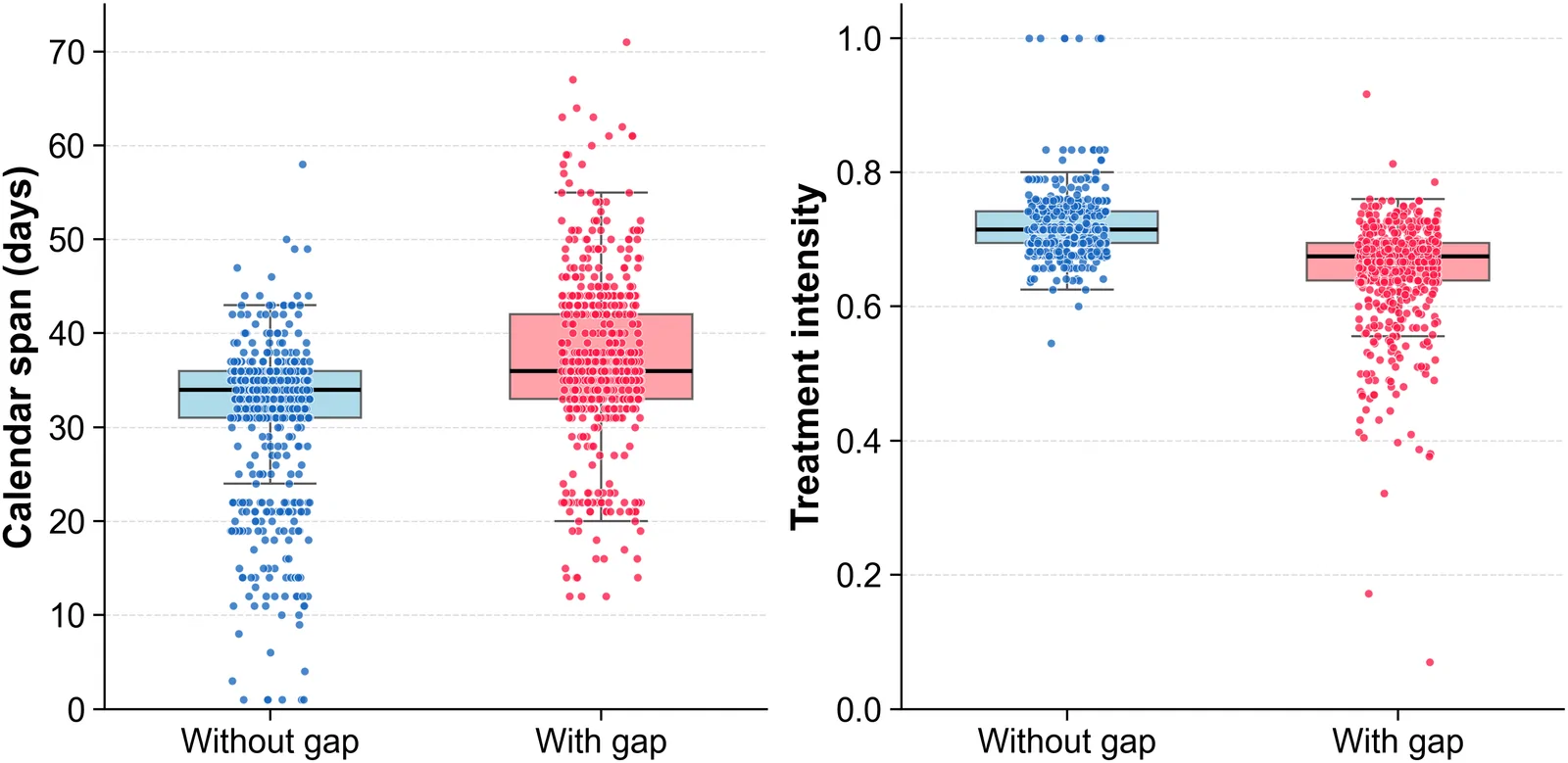
Box plots of calendar span (days) and treatment intensity (treatment days ÷ calendar span) comparing treatment episodes with versus without calendar-defined missed-workday gap. Individual episode values are shown as jittered points; boxes indicate interquartile range with median line.

**Figure 4:**
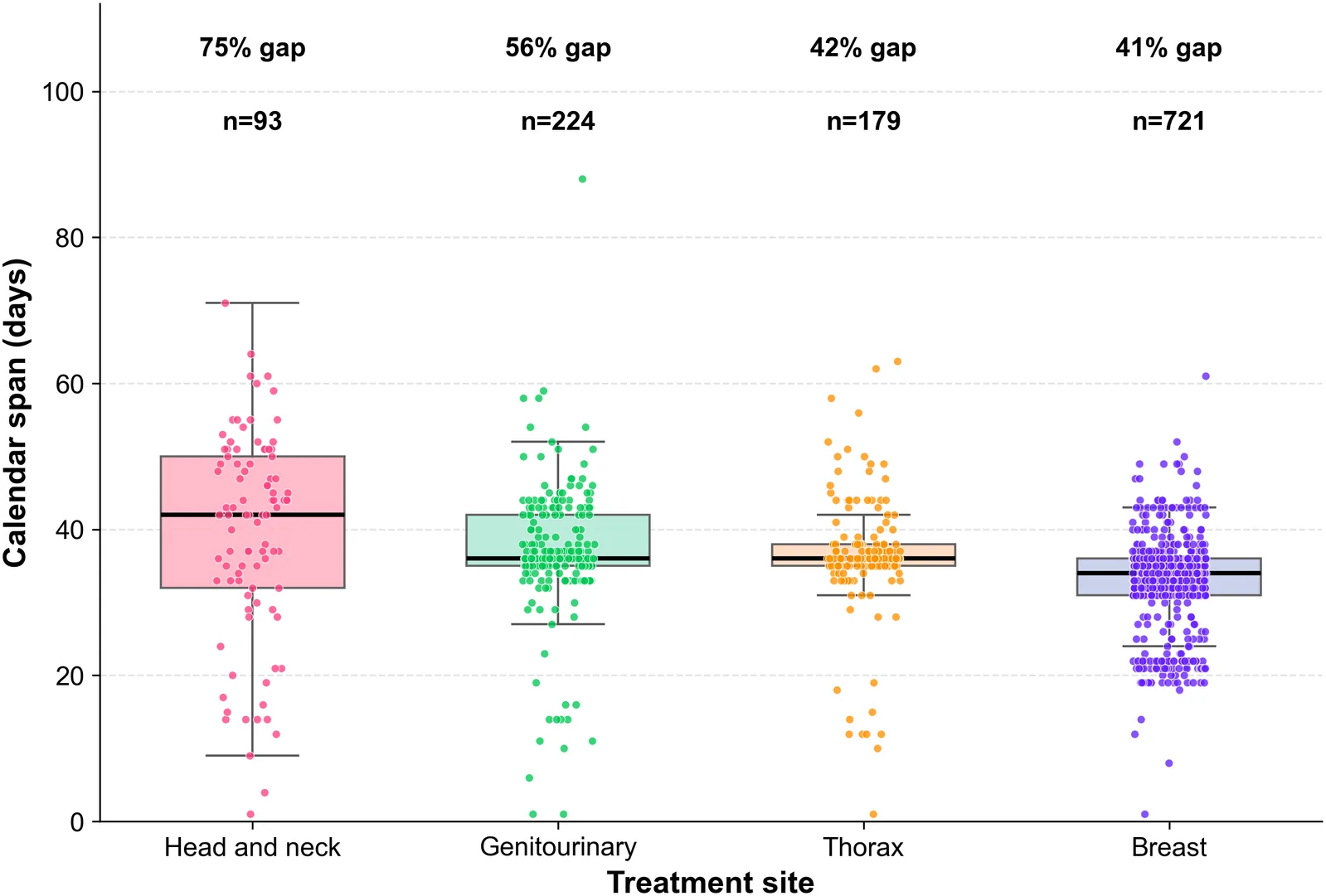
Box plots of calendar span by treatment site for prespecified subgroups with at least 50 episodes. Annotations show stratum size and calendar-defined missed-workday gap rate.

### Course phase (exploratory)

The early/middle/late analysis is exploratory. Phase thirds used the completed fraction index, so late-phase observations require course continuation. Episode-level phase analyses used each treatment episode as the unit of description. Among all 1,342 episodes, the proportion experiencing at least one calendar-defined missed-workday gap was 18.3% (95% CI, 16.4–20.5%) in the early course third, 21.2% (19.1–23.4%) in the middle third, and 23.0% (20.9–25.4%) in the late third; these repeated within-episode phase indicators are presented descriptively only (no independent-group trend test). At the gap level, workday-deficit frequencies by course phase in the full analytic cohort are shown in **Figure 5** (948 gap events: early 267, middle 309, late 372). Confirmatory gap-level inference used binomial GEE with episode-level clustering, logit link, exchangeable working correlation, and robust standard errors, after excluding episodes with ≤8 treatment days (n=29,763 inter-fraction intervals; 1,323 episode clusters). Relative to the early third, the late third showed higher odds of a qualifying gap (odds ratio [OR] 1.31; 95% CI 1.12–1.54; P = 0.0009), whereas the middle-versus-early contrast was smaller (OR 1.12; 95% CI 0.95–1.31; P = 0.169). Crude gap-level incidence in the confirmatory population was 2.8%, 3.1%, and 3.6% in the early, middle, and late thirds, respectively. After additional adjustment for site, calendar year, and intent, the late-versus-early OR was 1.32 (95% CI 1.12–1.57; P = 0.001) Among patients with calendar-defined missed-workday gap, maximum workday deficit by highest severity category is illustrated in **Figure 6**.

**Figure 5:**
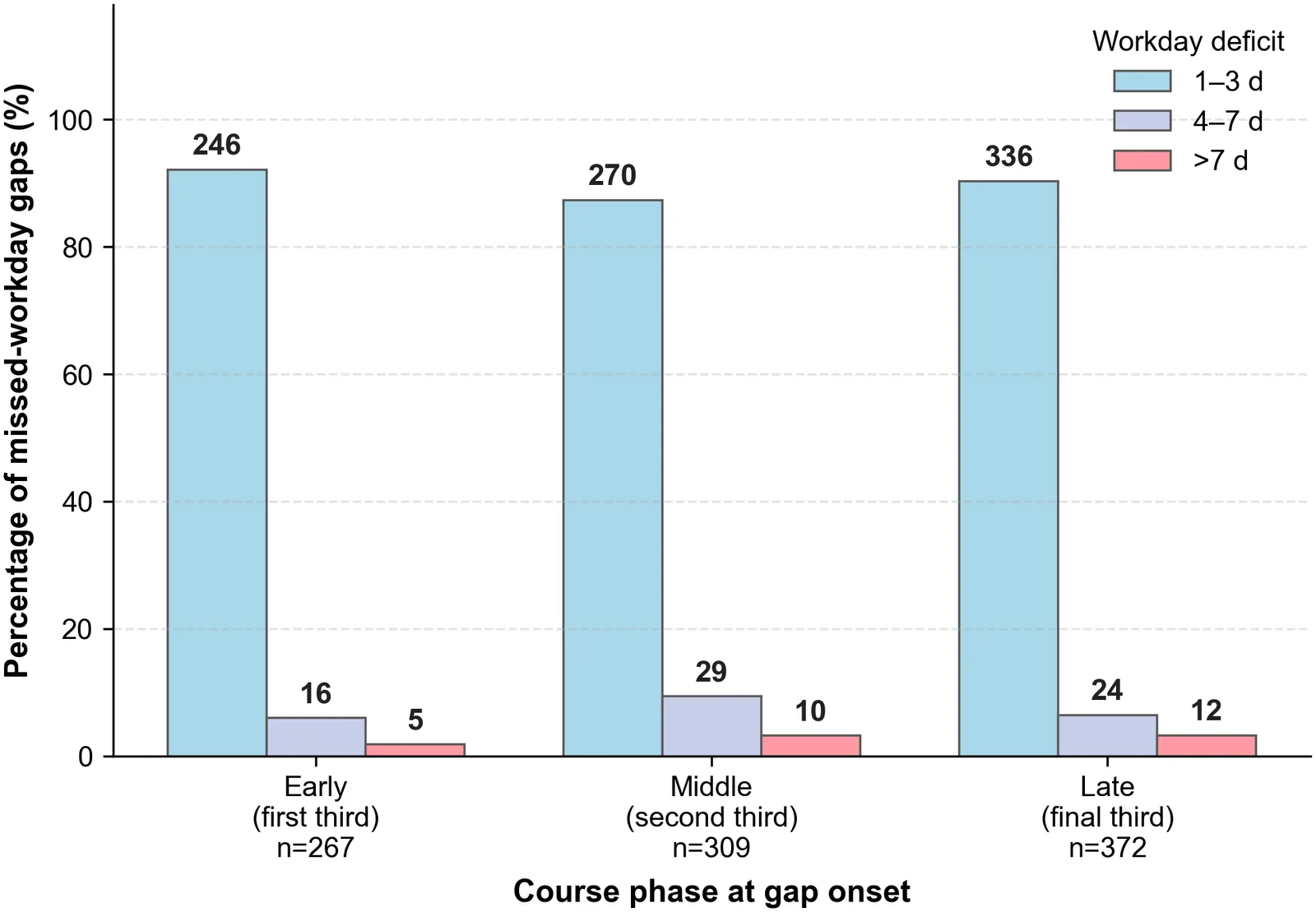
Frequency of workday-deficit categories (1–3, 4–7, >7 days) among calendar-defined missed-workday gaps, stratified by early, middle, and late course phase (exploratory descriptive display). Numbers above bars are gap counts; n beneath each phase is the number of gaps in that phase. Descriptive gap frequencies are shown for the full analytic cohort (948 gap events: early 267, middle 309, late 372); the confirmatory phase GEE excludes episodes with ≤8 treatment days (939 gap events: early 264, middle 306, late 369).

**Figure 6:**
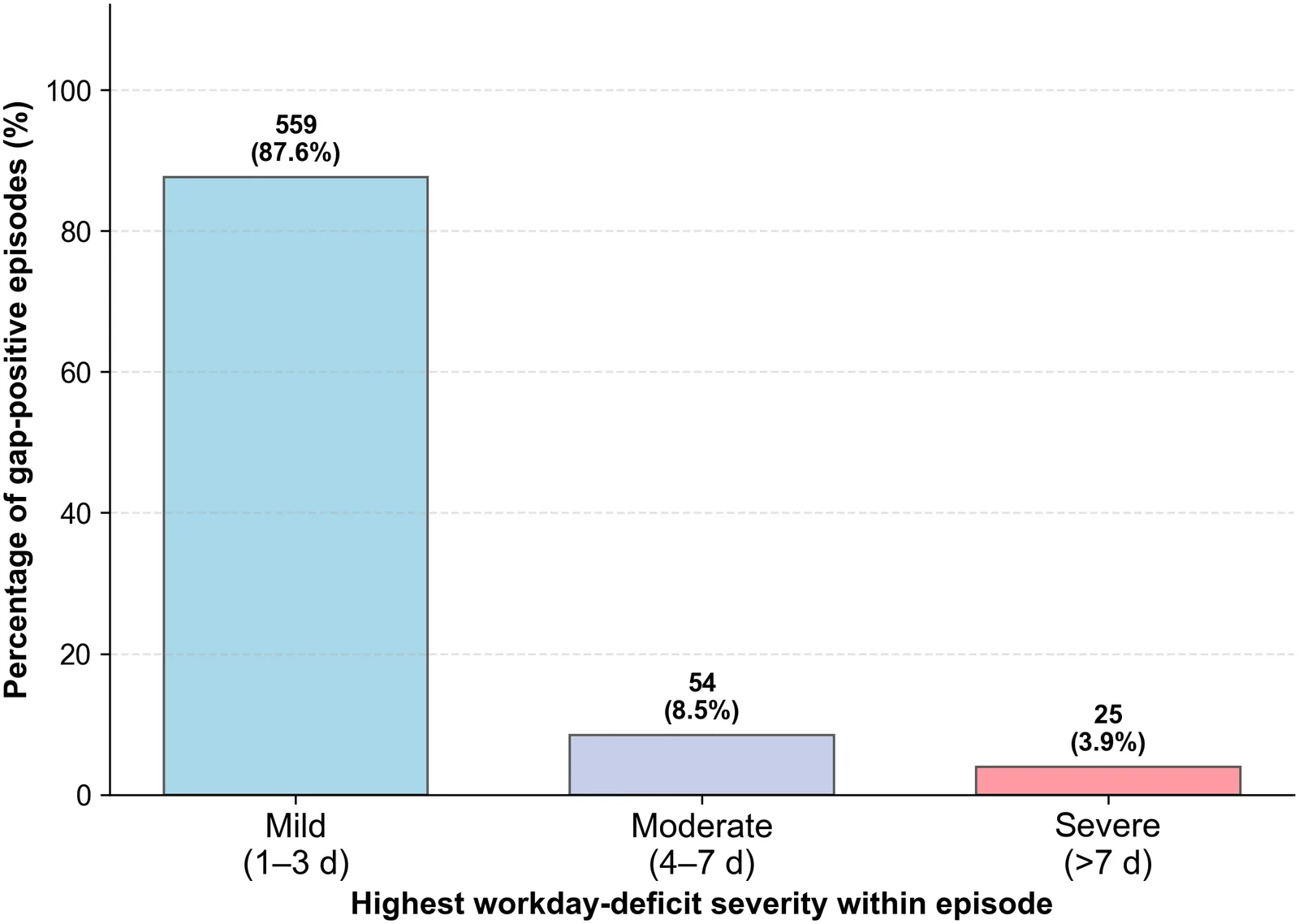
Frequency of maximum workday-deficit severity among gap-positive treatment episodes (mild 1–3 days, moderate 4–7 days, severe >7 days).

### Stratification by treatment site (≥50 episodes for reporting)

Calendar-defined missed-workday gap rates differed significantly across treatment sites (chi-square P<0.001; **Table 3**; **Figure 7**). Head-and-neck episodes had the highest point estimate (75.3%; 95% CI, 65.6–82.9%; n=93). Anatomic treatment site and disease-category mappings are distinct: breast-site episodes (n=721) are not identical to breast-cancer disease-category episodes (n=880), because some patients with a breast-cancer diagnosis received radiotherapy to non-breast anatomic sites. In exploratory disease-category analyses (prespecified ≥50-episode reporting threshold), calendar-defined missed-workday gap rates were 41.0% for breast cancer (n=880) and 56.2% for prostate cancer (n=201). Opportunity-adjusted analyses showed 5.63 (patient-clustered bootstrap 95% CI 4.76–6.52) gaps per 100 inter-fraction opportunities for head and neck versus 2.63 (2.36–2.92) for breast, 3.43 (2.92–3.99) for genitourinary, and 2.45 (1.99–2.92) for thorax. Patient-clustered Poisson GEE with opportunity offset (**Table 4**) yielded an adjusted incidence rate ratio (IRR) of 2.99 (95% CI 2.18–4.11; P<0.001) for head and neck versus breast. Full site-specific opportunity-adjusted estimates are shown in **Supplementary eTable 2**.

**Table 3:** Calendar-defined missed-workday gap by treatment site (prespecified subgroups with ≥50 episodes).

Site	n	Calendar-defined missed-workday gap, % (95% CI)	Calendar span, median, d	Treatment intensity
Breast	721	41.3 (37.8–45.0%)	34	0.694
Genitourinary	224	55.8 (49.3–62.2%)	36	0.694
Thorax	179	41.9 (34.9–49.2%)	36	0.694
Head and neck	93	75.3 (65.6–82.9%)	42	0.667

Rates are reported with Wilson score 95% CIs.

**Figure 7:**
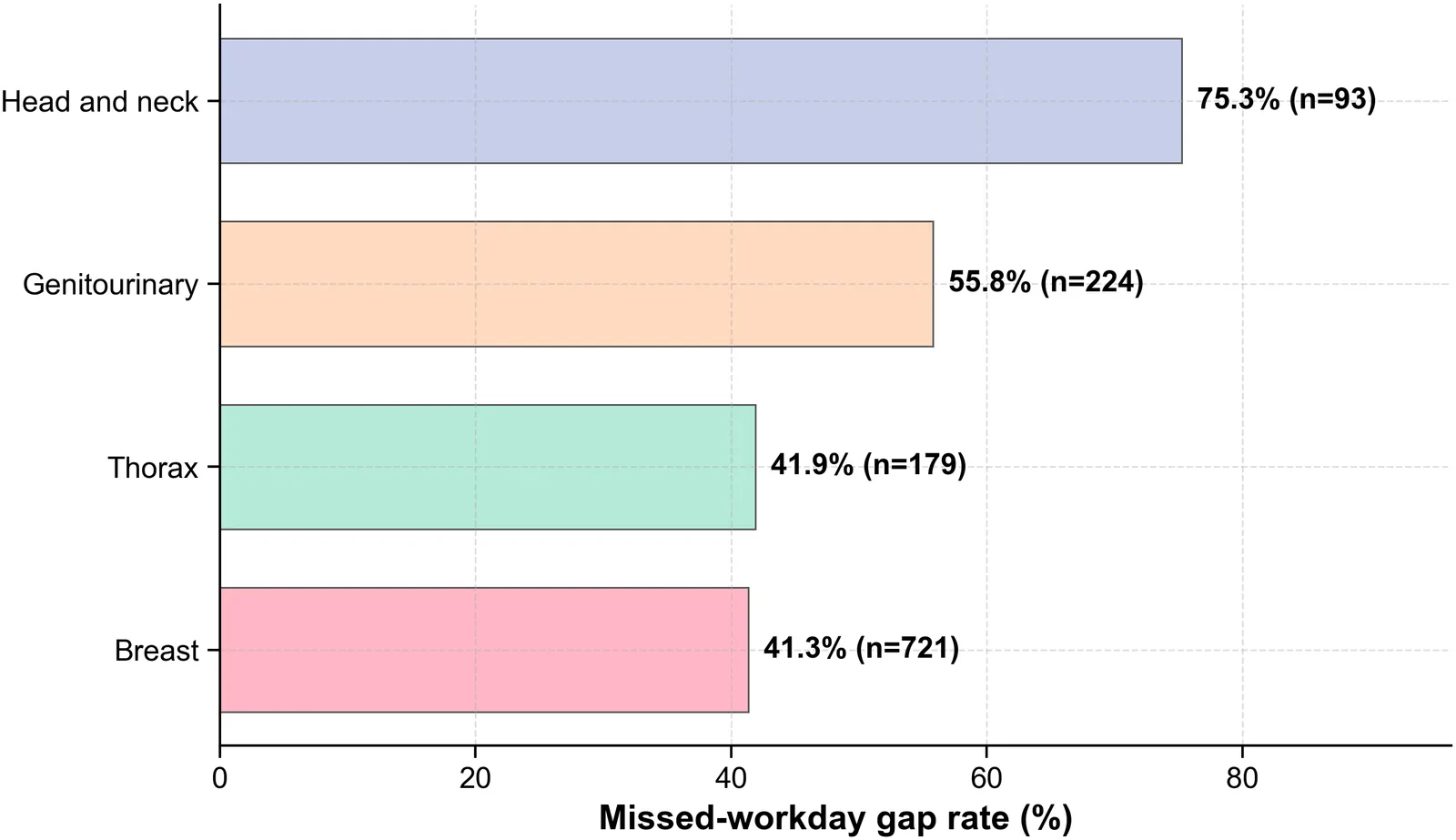
Calendar-defined missed-workday gap rate (%) by treatment site for prespecified subgroups with at least 50 episodes.

**Table 4:** Patient-clustered Poisson GEE for gap counts with log (inter-fraction opportunities) offset (reference: breast; exchangeable working correlation; robust standard errors; covariates: age, sex, intent, calendar year).

Variable	Adjusted IRR	95% CI	P value
Head and neck vs breast	2.99	2.18–4.11	<0.001
Genitourinary vs breast	1.71	1.23–2.37	0.001
Thorax vs breast	1.04	0.81–1.34	0.76
Other sites vs breast	2.21	1.67–2.93	<0.001
Age (per year)	1.00	0.99–1.01	0.99
Male sex	0.83	0.64–1.08	0.17
Palliative intent	1.14	0.78–1.66	0.51
Year 2022 vs 2021	1.39	1.11–1.74	0.004
Year 2023 vs 2021	1.30	1.00–1.69	0.054
Year 2024 vs 2021	1.86	1.43–2.42	<0.001
Year 2025 vs 2021	1.80	1.29–2.51	<0.001

Binomial GEE with treatment days is sensitivity only (**Supplementary eTable 12d**).

### Additional descriptive and sensitivity analyses

#### Weekend and holiday treatment

Treatment delivered on a weekend or statutory holiday occurred in 606 episodes (45.0%), with a median of 2 holiday treatment day(s) among those treated on rest days.

#### Gap prevalence by intent and calendar year

Gap prevalence was similar in radical/curative episodes (47.5% [44.7–50.2%]; n=1,266) and palliative episodes (48.7% [37.8–59.7%]; n=76) In the analytic cohort, episode-level prevalence by first-treatment year was 48.4% (2021), 49.7% (2022), 39.5% (2023), 50.8% (2024), and 53.7% (2025). Excluding 2022 yielded 46.8% (43.7–49.9%); excluding 2021–2022 yielded 46.3% (42.9–49.9%). Intent- and year-stratified estimates are summarized in **Supplementary eTables 3** and **4**; confirmatory and adjusted course-phase models are summarized in **Supplementary eTable 5**; denominators and splitting accounting are summarized in **Supplementary eTable 6**.

#### Course-splitting sensitivity

Among institutional-database–linkable patients, episode-level gap prevalence was 48.2% without splitting and 47.5% under the primary splitting rule (28 timelines flagged; net +23 in-window episodes). Across alternative gap thresholds of 30, 45, and 60 days, with or without disease-specific fraction caps, prevalence remained 47.2–47.9% (**Supplementary eTable 7**). Manual chart concordance was 25/28 (89.3%); the three discordant timelines retained original algorithmic episode assignments in the analytic cohort. Excluding those three patients left prevalence 47.5% (44.8–50.1%; 634/1,336); after collapsing the one over-split timeline, prevalence was 47.6% (44.9–50.3%; 638/1,341).

#### Tumor priority and A–D grades

Using locally adapted RCR-inspired categories, Category 1 episodes had the highest gap prevalence (79.3%; 95% CI, 67.2–87.7%; n=58) and the largest share of A/B grades (46/58, 79.3%), whereas Category 2 episodes (n=1,184) had a 46.0% gap prevalence with 61/1,184 (5.2%) graded A/B (**Supplementary eTables 8a, b**). Overall, 112/1,342 episodes (8.3%) were graded A or B. Compensation delivery was not assessed from MOSAIQ records; grades are audit flags under locally adapted RCR-inspired categories.

#### Same-day multi-session patterns (compensation proxy)

Using MOSAIQ timestamps and a ≥180-minute separation to define distinct same-day sessions, 3/1342 analytic episodes (0.2% (0.1–0.7%)) contained ≥1 multi-session day. Among radical episodes with ≥1 calendar-defined gap, the corresponding figure was 0.2% (0.0–0.9%). Multi-session prevalence was similar between episodes with versus without gaps (P = 1.000; **Supplementary eTable 9**). Same-day multi-session days were identified from MOSAIQ timestamps without chart review of compensation intent. Complementary non-treatment calendar days within the observed episode span (calendar span-treatment days), including planned weekend and holiday rest (median 11.0 days; 15.8% of episodes with ≥1 long-holiday-only rest interval) are summarized in **Supplementary eTable 10**. This quantity is a descriptive calendar span residual rather than true overall-treatment-time prolongation relative to a prescribed expected completion date. Keyword dictionaries, site-collapse rules, mapping concordance, course-splitting worked examples, short-course phase handling, model diagnostics (VIF/missingness), and specificity sampling status are provided in **Supplementary eTables 11a–h**.

#### Recurrent operational burden

The primary episode-level endpoint classified any ≥1 gap. Of 1,342 episodes, 704 had 0 gaps, 417 had exactly 1, and 221 had ≥2 (16.5% overall; 34.6% of the 638 gap-positive episodes). Among gap-positive episodes, the median number of gaps was 1 (IQR 1–2) and the median cumulative missed workdays was 1 (IQR 1–2) (**Supplementary eTable 12a**).

### Multivariable site analysis (opportunity-offset poisson GEE)

In the primary multivariable analysis, patient-clustered Poisson GEE for gap counts with log(inter-fraction opportunities) as an offset, adjusting for age, sex, treatment intent, and calendar year (n=1,248 episodes; 1,230 patient clusters; missing age and single-day episodes excluded), showed higher rates for head-and-neck versus breast (IRR 2.99; 95% CI 2.18–4.11; P<0.001), as did genitourinary (IRR 1.71; 95% CI 1.23–2.37; P = 0.001) and other-site episodes (IRR 2.21; 95% CI 1.67–2.93; P<0.001). Thoracic episodes showed an IRR close to 1 relative to breast (IRR 1.04; 95% CI 0.81–1.34; P = 0.76). Age was missing for 90 episodes; episodes with versus without recorded age had gap prevalence 47.0% (588/1,252) versus 55.6% (50/90) (chi-square P = 0.142; **Supplementary eTable 12b**). For the three discordant course-splitting timelines, excluding those patients left prevalence 47.5% (44.8–50.1%; 634/1,336), and collapsing the one over-split timeline left prevalence 47.6% (44.9–50.3%; 638/1,341) (**Supplementary eTable 12c**). In a sensitivity binomial GEE (age, sex, treatment days, intent), the head-and-neck odds ratio was 4.81 (95% CI 2.52–9.18; **Supplementary eTable 12d**).

## Discussion

In this linked cohort of 1,319 patients (1,342 treatment episodes) treated between June 2021 and December 2025, nearly half of episodes (47.5%; patient-level 48.0%) had at least one calendar-defined missed-workday gap after excluding routine weekends and statutory holidays. This burden is within the range reported in multicenter interruption audits (8–10). These gaps are operational indicators of institutional workdays without recorded treatment and are distinct from clinically confirmed unplanned interruptions. Within the same date window, unlinkable MOSAIQ patients had a gap prevalence of 51.7% versus 48.2% among linked patients (absolute difference −3.4 percentage points, 95% CI −11.2 to 4.4), arguing against major linkage-driven selection bias although unmeasured differences remain possible. The analyzed accelerator cohort was breast-predominant (721/1,342 episodes, 53.7%) because of preferential machine allocation; overall prevalence therefore reflects this case mix rather than a center-wide department estimate.

Chart review of algorithm-positive gaps clarifies the practical meaning of this operational burden: maintaining a continuous institutional-workday schedule is difficult in routine radiotherapy. Among 100 algorithm-positive gaps, 92 were labeled unplanned relevant under the documentation-based rule (17 documented department/equipment downtime; 75 patient-factor when no stop-treatment note existed; 8 planned/appropriate holiday-adjacent arrangements). Patient-factor labels may group acute toxicity, travel burden, competing appointments, or incomplete documentation rather than chart-confirmed refusal. Calendar-aware metrics can be automated from MOSAIQ delivery logs for quality dashboards paired with RCR-priority grading and alerts when Category 1 patients exceed workday-deficit thresholds (5, 7, 14). Because patient-factor labels outnumbered documented stoppages in the reviewed subsample, alerts may usefully trigger patient-support workflows (reminders, prompt toxicity review, transportation assistance, early contact after missed visits) while documented machine or departmental downtime is investigated when present.

The main methodologic feature of this study is the calendar-aware interruption framework combined with re-treatment episode splitting. Without splitting merged re-treatment timelines, calendar span and workday-deficit metrics can be spuriously inflated by multi-year gaps between courses. Conventional analyses that count all non-treatment calendar days may substantially overestimate quality gaps in departments where Saturday–Sunday and national holidays are standard rest days (5, 7). By distinguishing routine rest gaps from missing workdays and separating re-treatment episodes, we report an operational metric suited to RCR-oriented quality assurance. Treatment delivered on a weekend or statutory holiday occurred in 45.0% of episodes; without explicit scheduling or compensation documentation this observation is consistent with, but does not prove, continuity-oriented departmental scheduling during statutory rest periods (5, 8). Complementary non-treatment calendar days within the observed episode span (including planned weekend/holiday rest) are reported in **Supplementary eTable 10** and are best read as a descriptive calendar-span residual rather than true overall-treatment-time prolongation relative to a prescribed expected completion date.

Gap rates differed substantially by anatomic site. Head-and-neck patients exhibited the highest calendar-defined missed-workday gap rate (75.3%; 95% CI, 65.6–82.9%; n=93 among site subgroups meeting the ≥50-episode reporting threshold), and this association persisted after opportunity-offset Poisson GEE adjustment including calendar year (IRR 2.99 vs breast; **Table 4**); binomial GEE with treatment days was consistent in direction (OR 4.81). The wide confidence interval reflects the limited head-and-neck stratum size and supports cautious interpretation. This pattern is consistent with geometric complexity and adaptive-planning considerations in head-and-neck radiotherapy (22, 23). Genitourinary episodes, the largest non-breast stratum (n=224), showed intermediate burden (55.8%; 95% CI, 49.3–62.2%). Breast sites demonstrated a lower rate (41.3%; 95% CI, 37.8–45.0%), which may reflect shorter schedules and greater schedule resilience, although prolonged OTT has been associated with quality-of-care concerns in breast cancer cohorts (15). Site-specific estimates are associations after incomplete adjustment for stage, technique, systemic therapy, performance status, toxicity, and social factors.

The early/middle/late analysis is exploratory. Episode-level phase analyses showed a descriptive increase in the proportion of episodes with at least one calendar-defined missed-workday gap from the early (18.3%) to late (23.0%) course third; these repeated within-episode indicators were not evaluated with independent-group trend statistics. Gap-level incidence increased modestly from 2.8% early to 3.6% late; clustered GEE showed higher odds late versus early (OR 1.31; 95% CI 1.12–1.54; P = 0.0009), and 1.32 (1.12–1.57) after adjustment for site, year, and intent. Possible contributors include cumulative toxicity, equipment constraints, or patient fatigue (11, 19). Many first gap events still occurred in the early course phase, which supports attention to scheduling safeguards at treatment initiation (12, 13).

During portions of the study interval, mainland China implemented COVID-19 control measures. At our institution, radiotherapy patients were subject to continuity-oriented policies intended to limit disruption; although isolated patients experienced brief workday gaps, primary analyses were not stratified by pandemic era. International reports have documented variable operational gap burden during public-health emergencies (24–27), and unmeasured pandemic-related contributors remain possible in this single-center audit.

Limitations include the single-institution retrospective design, lack of oncologic outcomes (local control, survival), dependence on MOSAIQ documentation, heuristic course splitting (25/28 chart concordance; stable prevalence across sensitivity thresholds), and incomplete clinical covariate adjustment. Although gap prevalence did not differ significantly between episodes with versus without recorded age, absence of statistical significance does not establish that missingness was uninformative, and complete-case multivariable analyses may have introduced some degree of selection bias. CBCT/QA sessions were excluded using structured MOSAIQ fields; compensation delivery and biologically effective dose recovery were not modeled. Chart review applied documentation-based cause labels to algorithm-positive gaps only and was performed by a single reviewer; no independent second assessment or inter-rater agreement was obtained. The 92.0% unweighted UR proportion is not a clinical positive predictive value; patient-factor UR labels remain rule-based classifications when no department stop-treatment note existed and were not independently confirmed as patient-related or clinically inappropriate interruptions. Calendar-defined gaps are operational QA labels rather than substitutes for individualized compensation decisions. Planned long holidays may still prolong OTT despite exclusion from the operational endpoint. The cohort reflects breast-predominant machine selection and partial overlap with COVID-19 control periods without era-stratified primary analyses. External validation across institutions, machines, and holiday policies is needed.

## Conclusion

Calendar-defined missed-workday gaps affected nearly half of episodes in this breast-predominant accelerator cohort and should be read as an operational delivery audit rather than confirmation of unplanned clinical interruptions. Head-and-neck sites carried the highest burden after opportunity adjustment; an exploratory late-course signal was modest in absolute terms. Documentation-based chart review suggests that residual missed workdays often lack a department stop-treatment note. Calendar-aware auditing may support departmental monitoring; priority-based compensation remains a proposed application rather than an outcome demonstrated here.

## Data Availability

De-identified aggregate data and analysis scripts may be shared upon reasonable request, subject to institutional approval and applicable privacy regulations. Individual-level raw delivery records cannot be deposited publicly.

## References

[1] Withers HR Taylor JM Maciejewski B . The hazard of accelerated tumor clonogen repopulation during radiotherapy. Acta Oncol. (1988) 27:131–46. doi: 10.3109/02841868809090333 3390344

[2] Petereit DG Sarkaria JN Chappell R Fowler JF Hartmann TJ Kinsella TJ . The adverse effect of treatment prolongation in cervical carcinoma. Int J Radiat Oncol Biol Phys. (1995) 32:1301–7. doi: 10.1016/0360-3016(94)00635-x 7635769

[3] Bese NS Hendry JH Jeremic B . Effects of prolongation of overall treatment time due to unplanned interruptions during radiotherapy of different tumor sites and practical methods for compensation. Int J Radiat Oncol Biol Phys. (2007) 68:654–61. doi: 10.1016/j.ijrobp.2007.03.010 17467926

[4] González Ferreira JA Jaén Olasolo J Azinović I Jeremic B . Effect of radiotherapy delay in overall treatment time on local control and survival in head and neck cancer: Review of the literature. Rep Pract Oncol Radiother. (2015) 20:328–39. doi: 10.1016/j.rpor.2015.05.010 26549990 PMC4597087

[5] Royal College of Radiologists . The Timely Delivery of Radical Radiotherapy: Guidelines for the Management of Unscheduled Treatment Interruptions. 4th Ed. London: Royal College of Radiologists (2019).

[6] James ND Williams MV Summers ET Jones K Cottier B . The management of interruptions to radiotherapy in head and neck cancer: an audit of the effectiveness of national guidelines. Clin Oncol (R Coll Radiol). (2008) 20(8):599–605. doi: 10.1016/j.clon.2008.05.003 18555671

[7] Dale RG Hendry JH Jones B Robertson AG Deehan C Sinclair JA . Practical methods for compensating for missed treatment days in radiotherapy, with particular reference to head and neck schedules. Clin Oncol (R Coll Radiol). (2002) 14:382–93. doi: 10.1053/clon.2002.0111 12555877

[8] Pozo G Pérez-Escutia MA Ruíz A Ferrando A Milanés A Cabello E . Management of interruptions in radiotherapy treatments: Adaptive implementation in high workload sites. Rep Pract Oncol Radiother. (2019) 24:239–44. doi: 10.1016/j.rpor.2019.02.003 30858768 PMC6395853

[9] Fundowicz M Aguiar A de Castro CL Torras MG Deantonio L Konstanty E . Multicentre clinical radiotherapy audit in rectal cancer: Results of the IROCA project. Radiat Oncol. (2020) 15:208. doi: 10.1186/s13014-020-01648-7 32854730 PMC7453535

[10] de la Vega JM Ríos B Del Río JT Guerrero R Castillo I Guirado D . Management of interruptions to fractionated radiotherapy treatments: Four and a half years of experience. Phys Med. (2016) 32:1551–8. doi: 10.1016/j.ejmp.2016.11.108 27890566

[11] Giddings A . Treatment interruptions in radiation therapy for head-and-neck cancer: Rates and causes. J Med Imaging Radiat Sci. (2010) 41:222–9. doi: 10.1016/j.jmir.2010.08.002 31051883

[12] Ohri N Rapkin BD Guha D Haynes-Lewis H Guha C Kalnicki S . Predictive clinical model for interruption of radiotherapy. PLoS One. (2016) 11:e0165553. doi: 10.1371/journal.pone.0165553 27788254 PMC5082923

[13] Ohri N Rapkin BD Guha C Kalnicki S Garg M . Radiation therapy noncompliance and clinical outcomes in an urban academic cancer center. Int J Radiat Oncol Biol Phys. (2016) 95:563–70. doi: 10.1016/j.ijrobp.2016.01.043 27020104

[14] Hendry JH Bentzen SM Dale RG Fowler JF Wheldon TE Jones B . A modelled comparison of the effects of using different ways to compensate for missed treatment days in radiotherapy. Clin Oncol (R Coll Radiol). (1996) 8:297–307. doi: 10.1016/s0936-6555(05)80715-0 8934049

[15] Suryanegara FDA Rokhman MR Ekaputra E de Jong LA Setiawan D de Bock GH . Facing problems in radiotherapy for breast cancer patients in Yogyakarta, Indonesia: A cohort retrospective study. Cancer Med. (2023) 12:8851–9. doi: 10.1002/cam4.5634 36680328 PMC10134354

[16] Kwong DL Sham JS Chua DT Choy DT Au GK Wu PM . The effect of interruptions and prolonged treatment time in radiotherapy for nasopharyngeal carcinoma. Int J Radiat Oncol Biol Phys. (1997) 39:703–10. doi: 10.1016/s0360-3016(97)00339-8 9336153

[17] Kim MS Sheridan M Rajaraman M Hollenhorst H Caissie A Mahmoud-Ahmed A . The impact of national holidays on postoperative radiotherapy of squamous cell carcinoma of the head and neck. Clin Transl Radiat Oncol. (2023) 42:100668. doi: 10.1016/j.ctro.2023.100668 37608964 PMC10440354

[18] Xu C Yang KB Feng RJ Chen L Du XJ Mao YP . Radiotherapy interruption due to holidays adversely affects the survival of patients with nasopharyngeal carcinoma: A joint analysis based on large-scale retrospective data and clinical trials. Radiat Oncol. (2022) 17:36. doi: 10.1186/s13014-022-02006-5 35183221 PMC8858542

[19] Lindberg RD Jones K Garner HH Jose B Spanos WJ Bhatnagar D . Evaluation of unplanned interruptions in radiotherapy treatment schedules. Int J Radiat Oncol Biol Phys. (1988) 14:811–5. doi: 10.1016/0360-3016(88)90103-4 3350733

[20] Khalil AA Bentzen SM Bernier J Saunders MI Horiot JC Van Den Bogaert W . Compliance to the prescribed dose and overall treatment time in five randomized clinical trials of altered fractionation in radiotherapy for head-and-neck carcinomas. Int J Radiat Oncol Biol Phys. (2003) 55:568–75. doi: 10.1016/s0360-3016(02)03790-2 12573743

[21] von Elm E Altman DG Egger M Pocock SJ Gøtzsche PC Vandenbroucke JP . The strengthening the reporting of observational studies in epidemiology (STROBE) statement: Guidelines for reporting observational studies. PLoS Med. (2007) 4:e296. doi: 10.1371/journal.pmed.0040296 17941714 PMC2020495

[22] Barker JL Jr Garden AS Ang KK O'Daniel JC Wang H Court LE . Quantification of volumetric and geometric changes occurring during fractionated radiotherapy for head-and-neck cancer using an integrated CT/linear accelerator system. Int J Radiat Oncol Biol Phys. (2004) 59:960–70. doi: 10.1016/j.ijrobp.2003.12.024 15234029

[23] Nuyts S Bollen H Eisbruch A Strojan P Mendenhall WM Ng SP . Adaptive radiotherapy for head and neck cancer: Pitfalls and possibilities from the radiation oncologist's point of view. Cancer Med. (2024) 13:e7192. doi: 10.1002/cam4.7192 38650546 PMC11036082

[24] Xie C Wang X Liu H Bao Z Yu J Zhong Y . Clinical characteristics and outcomes of patients undergoing radiotherapy during the COVID-19 outbreak in Wuhan, China. JAMA Oncol. (2020) 6:1457–9. doi: 10.1001/jamaoncol.2020.2783 32729893 PMC7393581

[25] Gaudio E Ammar N Gunturkun F Akkus C Brakefield W Wakefield DV . Defining radiation treatment interruption rates during the COVID-19 pandemic: Findings from an academic center in an underserved urban setting. Int J Radiat Oncol Biol Phys. (2023) 116:379–93. doi: 10.1016/j.ijrobp.2022.09.073 36183931 PMC9584778

[26] Jereczek-Fossa BA Pepa M Zaffaroni M Marvaso G Bruni A Buglione di Monale Bastia M . COVID-19 safe and fully operational radiotherapy: An AIRO survey depicting the Italian landscape at the dawn of phase 2. Radiother Oncol. (2021) 155:120–2. doi: 10.1016/j.radonc.2020.09.049 33065185 PMC7553860

[27] Siavashpour Z Goharpey Z Mobasheri M . Radiotherapy based management during Covid-19 pandemic: A systematic review of presented consensus and guidelines. Crit Rev Oncol Hematol. (2021) 164:103402. doi: 10.1016/j.critrevonc.2021.103402 34214608 PMC8242203

