# Supplementary material for: Calendar-defined missed-workday gaps in radiotherapy: a site- and phase-stratified audit with linkage sensitivity analyses
Source: Front Oncol. 2026 Oct 7;16:1932765. doi: 10.3389/fonc.2026.1932765 (PMC13683152; doi:10.3389/fonc.2026.1932765)
Supplement: Supplementary file 1 [file Table1.docx]

**Supplementary Material**

**eTable 1.** Linked versus unlinked cleaned MOSAIQ patients with first treatment in the study window (June 2021–December 2025), without requiring clinical-database fields for the gap endpoint.

| **Characteristic** | **Linked (n=1,312)** | **Unlinked (n=180)** | **P value** |
| --- | --- | --- | --- |
| Patient-level gap prevalence, % (Wilson 95% CI) | 48.2% (45.6–51.0%) | 51.7% (44.4–58.9%) | 0.435* |
| Treatment days, median (IQR) | 25 (23–25) | 25 (23–26) | 0.001† |
| Calendar span, days, median (IQR) | 35 (32–37) | 36 (33–39) | 0.416† |
| Treatment intensity, median (IQR) | 0.694 (0.667–0.714) | 0.698 (0.667–0.722) | 0.018† |
| Maximum workday deficit, median (IQR) | 0 (0–1) | 1 (0–1) | 0.183† |

*Chi-square test. †Mann–Whitney U test comparing distributions (not a test of median equality alone); identical or near-identical medians may still yield P<0.05 when rank distributions differ (treatment-day means: 23.66 vs 22.76; intensity means: 0.681 vs 0.699). Linkage rate in window: 87.9% (1,312/1,492). Full cleaned MOSAIQ cohort irrespective of year (n=4,285): patient-level gap prevalence 55.1% (53.7–56.6%).

**eTable 2.** Episode-level prevalence versus opportunity-adjusted gap density by treatment site. For breast, genitourinary, thorax, and head-and-neck strata, gaps-per-100-opportunities confidence intervals are patient-clustered bootstrap 95% CIs. For smaller site strata marked ‡, Wilson intervals are shown for description only.

| **Treatment site** | **Episodes, n** | **Episode-level ≥1 gap, % (95% CI)** | **Inter-fraction opportunities, n** | **Gap events, n** | **Gaps per 100 opportunities (95% CI)** |
| --- | --- | --- | --- | --- | --- |
| Breast | 721 | 41.3% (37.8–45.0%) | 15,673 | 412 | 2.63 (2.36–2.92) |
| Genitourinary | 224 | 55.8% (49.3–62.2%) | 5,329 | 183 | 3.43 (2.92–3.99) |
| Thorax | 179 | 41.9% (34.9–49.2%) | 4,158 | 102 | 2.45 (1.99–2.92) |
| Head and neck | 93 | 75.3% (65.6–82.9%) | 2,237 | 126 | 5.63 (4.76–6.52) |
| Gynecologic‡ | 36 | 77.8% (61.9–88.3%) | 837 | 63 | 7.53 (5.93–9.51) |
| Intracranial‡ | 32 | 53.1% (36.4–69.1%) | 589 | 27 | 4.58 (3.17–6.59) |
| Extremity‡ | 23 | 30.4% (15.6–50.9%) | 339 | 10 | 2.95 (1.61–5.34) |
| Abdomen‡ | 23 | 47.8% (29.2–67.0%) | 444 | 16 | 3.60 (2.23–5.77) |
| Pelvis‡ | 6 | 66.7% (30.0–90.3%) | 89 | 6 | 6.74 (3.13–13.94) |
| Rectum‡ | 5 | 60.0% (23.1–88.2%) | 128 | 3 | 2.34 (0.80–6.66) |

Wilson 95% CIs. Opportunities = all inter-fraction intervals within episodes. Prespecified reporting threshold for main-text site tables was ≥50 episodes; sites below this threshold (‡) are shown here for completeness and should be interpreted cautiously. Head-and-neck episodes retained higher gap density per opportunity than breast, indicating that higher episode-level prevalence is not explained solely by longer courses.

**eTable 3.** Calendar-defined missed-workday gaps by treatment intent in the analytic linked cohort.

| **Treatment intent** | **Episodes, n** | **Episodes with ≥1 gap, n** | **Prevalence, % (95% CI)** |
| --- | --- | --- | --- |
| Palliative | 76 | 37 | 48.7% (37.8–59.7%) |
| Radical/curative | 1,266 | 601 | 47.5% (44.7–50.2%) |

Clinical implications of gaps differ by intent; palliative estimates are imprecise owing to limited n.

**eTable 4.** Episode-level gap prevalence by calendar year and pandemic-overlapping sensitivity analyses in the analytic linked cohort.

| **First-treatment year / sensitivity** | **Episodes, n** | **With ≥1 gap, n** | **Prevalence, % (95% CI)** |
| --- | --- | --- | --- |
| 2021 | 225 | 109 | 48.4% (42.0–54.9%) |
| 2022 | 338 | 168 | 49.7% (44.4–55.0%) |
| 2023 | 339 | 134 | 39.5% (34.5–44.8%) |
| 2024 | 319 | 162 | 50.8% (45.3–56.2%) |
| 2025 | 121 | 65 | 53.7% (44.9–62.4%) |
| Exclude 2022 | 1,004 | 470 | 46.8% (43.7–49.9%) |
| Exclude 2021–2022 | 779 | 361 | 46.3% (42.9–49.9%) |

**eTable 5.** Gap-level course-phase analysis with episode-clustered binomial GEE after excluding episodes with ≤8 treatment days (n=29,763 intervals; 1,323 episode clusters; logit link; exchangeable working correlation; robust standard errors). Unadjusted late versus early OR 1.31 (1.12–1.54), P=0.0009. In an exploratory model additionally adjusted for site, calendar year, and treatment intent, late versus early OR was 1.32 (1.12–1.57), P=0.001. Absolute crude rates were 2.8%/3.1%/3.6% (early/middle/late). Phase analyses are exploratory.

| **Course phase** | **Gaps, n** | **Gap events, n** | **Crude gap incidence, % (95% CI)** | **GEE OR vs early** | **GEE P** |
| --- | --- | --- | --- | --- | --- |
| Early third | 9,568 | 264 | 2.8% (2.4-3.1%) | 1.00 (ref) | — |
| Middle third | 9,933 | 306 | 3.1% (2.8-3.4%) | 1.12 (0.95-1.31) | 0.169 |
| Late third | 10,262 | 369 | 3.6% (3.3-4.0%) | 1.31 (1.12-1.54) | 0.0009 |

Episode-level proportions with ≥1 gap by phase (18.3%, 21.2%, 23.0%) are descriptive only because phase indicators are repeated within episodes; no independent-group Cochran–Armitage test is reported. Confirmatory inference relies on the clustered gap-level GEE below. Crude rates and GEE estimates use the short-course–excluded population.

**eTable 6.** Analysis denominators, course-splitting accounting, and patient- versus episode-level prevalence.

| **Metric** | **Value** |
| --- | --- |
| Unique patients in analytic cohort | 1,319 |
| Treatment episodes after splitting | 1,342 |
| Net additional episodes (episodes − patients) | 23 |
| Patients flagged by splitting algorithm | 28 |
| Episode counts among flagged patients (1 / 2 / 3 episodes) | 6 / 21 / 1 |
| Patient-level ≥1 gap | 633/1,319 (48.0% (45.3–50.7%)) |
| One-episode-per-patient sensitivity | 627/1,319 (47.5% (44.9–50.2%)) |
| Episode-level ≥1 gap (primary) | 638/1,342 (47.5% (44.9–50.2%)) |

Net additional episodes can be fewer than the number of patients flagged for splitting when some flagged timelines contribute only one retained episode after date-window filtering or when splits yield more than two segments in a minority of patients. Manual chart review of the 28 in-window flagged timelines showed concordance in 25/28 (89.3%).

**eTable 7.** Course-splitting sensitivity analyses among institutional-database–linkable MOSAIQ patients (episode retained if first treatment date in June 2021–December 2025).

| **Scenario** | **Timelines flagged** | **Patients (window)** | **Episodes (window)** | **Net extra episodes** | **Episode-level ≥1 gap % (95% CI)** | **Episodes with max WD deficit >7** |
| --- | --- | --- | --- | --- | --- | --- |
| No splitting (one timeline/patient) | 0 | 1,312 | 1,312 | 0 | 48.2% (45.6–51.0%) | 40 |
| Primary rule: gap≥45 d + fraction caps | 28 | 1,319 | 1,342 | 23 | 47.5% (44.9–50.2%) | 25 |
| Sensitivity: gap≥30 d + caps (always apply gap) | 29 | 1,319 | 1,343 | 24 | 47.4% (44.8–50.1%) | 24 |
| Sensitivity: gap≥30 d, no fraction caps | 26 | 1,319 | 1,340 | 21 | 47.6% (44.9–50.3%) | 27 |
| Sensitivity: gap≥45 d + caps (always apply gap) | 28 | 1,319 | 1,342 | 23 | 47.5% (44.9–50.2%) | 25 |
| Sensitivity: gap≥45 d, no fraction caps | 24 | 1,319 | 1,338 | 19 | 47.8% (45.1–50.4%) | 29 |
| Sensitivity: gap≥60 d + caps (always apply gap) | 27 | 1,319 | 1,341 | 22 | 47.6% (44.9–50.3%) | 26 |
| Sensitivity: gap≥60 d, no fraction caps | 21 | 1,318 | 1,335 | 17 | 47.9% (45.3–50.6%) | 31 |

Primary analysis uses gap≥45 days plus disease-specific fraction caps with the original review gate. Sensitivity scenarios force application whenever any inter-fraction gap meets the stated threshold. Gap prevalence remained stable across unsplit and alternative splitting rules.

**eTable 8a.** Calendar-defined missed-workday gaps by locally adapted RCR tumor-priority category.

| **Tumor priority (locally adapted RCR)** | **Episodes, n** | **≥1 gap % (95% CI)** | **Median max WD deficit** | **Grade A or B, n (%)** | **Max WD deficit >7, n** |
| --- | --- | --- | --- | --- | --- |
| Category 2 (intermediate) | 1,184 | 46.0% (43.2–48.9%) | 0 | 61 (5.2%) | 18 |
| Category 3 (lower) | 100 | 47.0% (37.5–56.7%) | 0 | 5 (5.0%) | 5 |
| Category 1 (high) | 58 | 79.3% (67.2–87.7%) | 1 | 46 (79.3%) | 2 |

**eTable 8b.** A–D clinical-significance grades combining priority and maximum workday deficit.

| **Clinical-significance grade** | **Episodes, n** | **% of analytic cohort** | **Median max WD deficit** |
| --- | --- | --- | --- |
| No calendar-defined gap | 704 | 52.5 | 0 |
| C (general) | 484 | 36.1 | 1 |
| D (low impact) | 42 | 3.1 | 1 |
| B (compensation / attention) | 68 | 5.1 | 3 |
| A (severe / priority-linked) | 44 | 3.3 | 7 |

Decision matrix: Grades A–D are assigned only to episodes with ≥1 calendar-defined missed-workday gap; episodes without such a gap are labeled “No calendar-defined gap” and do not receive an A–D grade. Among gap-positive episodes, grades combine locally adapted RCR priority with maximum workday deficit (A: priority-linked severe; B: compensation/attention; C: general; D: low impact). Regenerated counts: no-gap 704; A 44; B 68; C 484; D 42 (A–D sum = 638). Actual compensation delivery could not be determined from MOSAIQ alone and is proposed as a future application. In the analytic cohort, 112 episodes (8.3%) were graded A or B (A: 44; B: 68).

**eTable 9.** Same-day multi-session patterns on MOSAIQ timestamps (session gap ≥180 minutes).

| **Subgroup** | **Episodes, n** | **≥1 same-day multi-session day, n** | **Prevalence, % (95% CI)** |
| --- | --- | --- | --- |
| All analytic episodes | 1,342 | 3 | 0.2% (0.1–0.7%) |
| Episodes with ≥1 calendar-defined gap | 638 | 1 | 0.2% (0.0–0.9%) |
| Episodes without calendar-defined gap | 704 | 2 | 0.3% (0.1–1.0%) |
| Radical/curative | 1,266 | 3 | 0.2% (0.1–0.7%) |
| Palliative | 76 | 0 | 0.0% (0.0–4.8%) |
| Radical episodes with ≥1 gap | 601 | 1 | 0.2% (0.0–0.9%) |
| Head and neck | 93 | 0 | 0.0% (0.0–4.0%) |
| Breast | 721 | 1 | 0.1% (0.0–0.8%) |

Proxy only; chi-square P (gap vs no-gap)=1.000. Does not prove compensatory intent.

**eTable 10.** Non-treatment calendar days within the observed episode span (including planned weekend/holiday rest)

Complementary to the operational missed-workday-gap endpoint. Planned weekend/holiday rest can still prolong overall treatment time relative to an ideal continuous schedule and is summarized here without implying that such intervals are clinically harmless. The quantity formerly labeled “calendar extension” equals calendar span − treatment days and counts non-treatment calendar days within the observed episode; it is not true OTT prolongation versus a prescribed expected completion date. Values are median (IQR) unless noted.

| **Stratum** | **n** | **Calendar extension (d)** | **Operational missed workdays** | **Weekend/holiday rest days in gaps** | **Long-holiday-only calendar days** | **Episodes with long-holiday rest gap** |
| --- | --- | --- | --- | --- | --- | --- |
| All episodes | 1342 | 11.0 (9.0–12.0) | 0.0 (0.0–1.0) | 10.0 (8.0–11.0) | 0.0 (0.0–0.0) | 15.8% (13.9–17.8%) |
| Head and neck | 93 | 14.0 (9.0–18.0) | 2.0 (1.0–4.0) | 12.0 (8.0–15.0) | 0.0 (0.0–0.0) | 17.2% (10.9–26.1%) |
| Breast | 721 | 10.0 (8.0–11.0) | 0.0 (0.0–1.0) | 10.0 (8.0–11.0) | 0.0 (0.0–0.0) | 14.4% (12.0–17.2%) |
| Thorax | 179 | 11.0 (10.0–12.0) | 0.0 (0.0–1.0) | 11.0 (10.0–11.0) | 0.0 (0.0–0.0) | 17.9% (13.0–24.1%) |
| Genitourinary | 224 | 11.0 (10.0–13.0) | 1.0 (0.0–1.0) | 11.0 (10.0–12.0) | 0.0 (0.0–0.0) | 19.2% (14.6–24.9%) |
| Category 1 (high) | 58 | 15.0 (11.0–18.0) | 2.0 (1.0–3.0) | 13.0 (10.0–16.0) | 0.0 (0.0–3.0) | 25.9% (16.3–38.4%) |
| Category 2 (intermediate) | 1184 | 11.0 (9.0–12.0) | 0.0 (0.0–1.0) | 10.0 (8.0–11.0) | 0.0 (0.0–0.0) | 15.4% (13.4–17.5%) |
| Category 3 (lower) | 100 | 8.0 (5.0–12.0) | 0.0 (0.0–1.0) | 8.0 (4.8–11.2) | 0.0 (0.0–0.0) | 15.0% (9.3–23.3%) |

Footnote: Non-treatment calendar days = calendar span − treatment days. Operational missed workdays exclude routine weekend/holiday rest by definition. Planned calendar rest counts each non-treatment weekend or holiday day once (holiday-weekends are not double-counted). Long-holiday-only intervals contain no untreated workday and are excluded from the operational gap endpoint but contribute to non-treatment calendar days within the observed span.

**eTable 11 (Methods integrity)**
Keyword dictionaries, mapping concordance on the chart-review subsample, course-splitting worked examples, short-course phase handling, model diagnostics (VIF/missingness), specificity sampling status, and computational pipeline/software.

**eTable 11a.** Keyword dictionary for locally adapted RCR-inspired priority

Keyword rules were applied programmatically from diagnosis, site, and intent fields; categories are locally adapted and were not prospectively mandated RCR assignments.

| **Priority** | **Rule type** | **Keywords / logic (English gloss of Chinese EHR tokens)** |
| --- | --- | --- |
| Category 3 (lower) | Intent | Palliative intent |
| Category 3 (lower) | Benign/low-risk keywords | PVNS; meningioma; TAO/ophthalmopathy/Graves; synovitis; giant cell tumor; hyperthyroidism |
| Category 1 (high) | Rapid-repopulation keywords | nasopharyngeal carcinoma/nasopharyngeal tumor; oropharynx; laryngeal carcinoma/laryngeal tumor; hypopharynx; esophagus; small-cell lung cancer; nasal cavity/paranasal sinus; maxillary sinus carcinoma; gingival carcinoma; oral cavity carcinoma; tongue carcinoma; parotid carcinoma; hypopharynx; middle-ear carcinoma; olfactory neuroblastoma |
| Category 2 (intermediate) | Common radical histologies | breast/breast cancer; cervix; endometrium; vulva; rectum; prostate; lung cancer; glioma; bladder; renal pelvis/ureter/renal cancer; gastric cancer; lymphoma; thymus; sarcoma/osteosarcoma |
| Category 2 (intermediate) | Site fallback | If site contains head and neck/thorax/pelvis/gynecologic/genitourinary/abdomen and not otherwise classified → Category 2 |
| Category 2 (intermediate) | Default | Unclassified diagnoses default to Category 2 for retrospective audit (conservative for over-calling Category 1) |

**eTable 11b.** Site collapse dictionary

Site labels were collapsed programmatically from free-text and coded treatment-site fields to the analytic site categories used in the manuscript.

| **Source token (English gloss of Chinese EHR field)** | **Collapsed site category** |
| --- | --- |
| Breast | Breast |
| Genitourinary | Genitourinary |
| Thorax | Thorax |
| Head and neck | Head and neck |
| Gynecologic | Gynecologic |
| Intracranial | Intracranial |
| Abdomen | Abdomen |
| Extremity | Extremity |
| Rectum | Rectum |
| Pelvis / lumbosacral spine | Pelvis |

**eTable 11c.** Mapping concordance on chart-review subsample

Patients overlapping the locked chart-validation samples (positive-gap n=100 and/or split review): n=116. Priority concordance between the operational keyword algorithm and an independent clinician-style heuristic: 113/116 = 97.4% (92.7–99.1%). Site fields mapped to a known collapsed category in 116/116 (100.0%). Disagreements were mainly default-to-Category-2 vs histology-suggested Category 1, or ambiguous free-text diagnoses. Category 1 and Category 3 keyword lists were manually rechecked for clearly inappropriate assignments (including gingival malignancy and nasal NK/T-cell lymphoma terminology). Discordant free-text cases remain documented as illustrative disagreements rather than silent remapping of the locked analytic cohort.

**Illustrative disagreements (de-identified truncation).**

| **Algorithm** | **Heuristic** | **Heuristic basis** | **Diagnosis (truncated)** |
| --- | --- | --- | --- |
| Category 2 | Category 1 | HN-site malignant cue | Endometrial tumor, cervical lymph-node metastasis |
| Category 2 | Category 1 | Category 1 histology keyword | Other, gingival tumor |
| Category 2 | Category 1 | Category 1 histology keyword | Other, nasal NK/T-cell lymphoma |

**eTable 11d.** Course-splitting worked examples

Heuristic rules to keep separate courses from being merged when electronic course IDs are incomplete; chart concordance for flagged cases 25/28 (89.3%).

| **Example ID** | **Rule** | **Disease/site** | **Orig. days / segments** | **Explanation** |
| --- | --- | --- | --- | --- |
| EX-45d | ≥45-day inter-fraction gap | Illustrative/— | e.g. 25+20 → 2 | A ≥45-day untreated interval separates concatenated courses when electronic course IDs are unreliable. |
| EX-cap-breast | Breast fraction cap ≤33 | Breast/Breast | >33 → ≥2 | Timelines exceeding the institutional breast fraction ceiling are reviewed/split at the largest internal gap. |
| EX-cap-prostate | Prostate fraction cap ≤40 | Prostate/GU | >40 → ≥2 | Prostate courses use a higher cap than other digestive/GU sites (≤35). |
| EX-span60 | Calendar span >60 days → review | Illustrative/— | variable → review | Long calendar span triggers manual inspection even when daily gaps are <45 days. |
| SPL-01 | heuristic split | Nasopharyngeal carcinoma / head and neck | 51 → 2 | Algorithm-flagged split timeline (de-identified). |
| SPL-02 | heuristic split | Other head and neck / abdomen | 41 → 2 | Algorithm-flagged split timeline (de-identified). |
| SPL-03 | heuristic split | Endometrial carcinoma / head and neck | 108 → 4 | Algorithm-flagged split timeline (de-identified). |

**eTable 11e.** Short-course phase handling (text note; no numeric table).

Definition: short course = ≤8 treatment days. In the analytic cohort, 19 / 1,342 episodes (1.4%) met this definition. Confirmatory phase GEE excluded these episodes (n excluded = 19), reducing the analysis from 29,823 intervals / 1,336 clusters in the full gap set to 29,763 intervals / 1,323 episode clusters。 Descriptive early/middle/late episode-level percentages in the main text use the full cohort for continuity with prior reporting. Stereotactic/very short palliative regimens are therefore not interpreted as having stable course thirds. GEE specification: binomial family, logit link, exchangeable working correlation, robust/sandwich standard errors; ORs reported with 95% CIs.

**eTable 11f.** Model diagnostics (VIF and missingness)

Complete-case design matrix n=1,252. Age complete-case episodes: 1,252 (missing/zero-coded: 90). VIF≪5 suggests no concerning multicollinearity among listed covariates.

| **Covariate** | **VIF** |
| --- | --- |
| Age (per year) | 1.32 |
| Treatment days | 1.09 |
| Male sex | 3.49 |
| Palliative intent | 1.16 |
| Head and neck vs breast | 1.49 |
| Thorax vs breast | 1.16 |
| Genitourinary vs breast | 3.57 |
| Other sites vs breast | 1.28 |

Footnote: Complete-case design matrix n=1,252 (age missing/zero-coded: 90). Breast is the reference site category. All VIFs ≪5, indicating no concerning multicollinearity among covariates in the Table 4 specification. Moderately higher VIFs for male sex and genitourinary site reflect their known association in this cohort rather than problematic collinearity.

**eTable 11g.** Specificity sampling status (text note; no numeric table).

A negative-frame chart sample (algorithm-negative intervals) was not performed. Consequently sensitivity and specificity are not reported. In the positive-frame sample (n=100), UR=92 (17 confirmed department/equipment; 75 patient-factor labels assigned by a documentation-based rule) and PA=8 (planned/appropriate). The unweighted sample proportion classified as unplanned relevant under the prespecified adjudication rule was 92.0% (85.0–95.9%). Patient-factor labels followed a documentation-based rule that department-arranged stoppages are charted and should not be read as chart-confirmed refusal in every case. The manuscript retains the operational endpoint calendar-defined missed-workday gap and interprets cause mix as evidence that ideal continuous scheduling is hard to achieve, with patient-factor labels predominating among unplanned positives under a documentation-based rule.

**eTable 11h.** Computational pipeline and software (text note).

Analyses were implemented in Python with version-controlled scripts. Inter-fraction calendars used the Chinese mainland statutory holiday calendar (including adjusted workdays) via the Chinese calendar package (≥1.9). Cohort cleaning, episode splitting, opportunity-adjusted rates, clustered GEE, and table/figure exports were run from locked analytic scripts corresponding to the Path A revision outputs. Keyword dictionaries for disease category, site collapse, and locally adapted RCR-inspired priority are listed in eTables 11a–11b; mapping concordance on the chart-review subsample is in eTable 11c. No protected health information is embedded in the pipeline description.

**eTable 12a.** Recurrent operational gap burden at the episode level (primary endpoint remains any ≥1 gap).

| Gap-count category | Episodes, n | % of 1,342 | % of gap-positive (n=638) |
| --- | --- | --- | --- |
| 0 gaps | 704 | 52.5% | — |
| Exactly 1 gap | 417 | 31.1% | 65.4% |
| ≥2 gaps | 221 | 16.5% | 34.6% |
| Among gap-positive: median (IQR) gaps | 1 (1–2) |  |  |
| Among gap-positive: median (IQR) cumulative missed workdays | 1 (1–2) |  |  |

**eTable 12b**. Missing age and episode-level gap prevalence. Multivariable models used complete cases.

| Age availability | Episodes, n | Any gap, n | Prevalence (Wilson) | Chi-square P |
| --- | --- | --- | --- | --- |
| Age recorded | 1,252 | 588 | 47.0% (44.2–49.7%) | 0.142 |
| Age missing | 90 | 50 | 55.6% (45.3–65.4%) |  |

**eTable 12c**. Course-splitting concordance sensitivity (28 flagged timelines; concordance 25/28). Discordant cases: SPL-009 and SPL-010 under-split; SPL-020 over-split.

| Analysis set | Episodes, n | Any gap, n | Prevalence (Wilson) |
| --- | --- | --- | --- |
| Primary (algorithm retained) | 1,342 | 638 | 47.5% (44.9–50.2%) |
| Exclude 3 discordant patients | 1,336 | 634 | 47.5% (44.8–50.1%) |
| Collapse over-split only | 1,341 | 638 | 47.6% (44.9–50.3%) |
| Patient-level any gap | 1,319 | 633 | 48.0% (45.3–50.7%) |

**eTable 12d**. Sensitivity analysis: patient-clustered binomial GEE for any gap (former main-text Table 4 specification; age, sex, treatment days, intent; n=1,248 episodes; 1,230 patient clusters). Main-text Table 4 now reports opportunity-offset Poisson IRR with calendar year.

| Variable | Adjusted OR | 95% CI | P value |
| --- | --- | --- | --- |
| Head and neck vs breast | 4.81 | 2.52–9.18 | <0.001 |
| Genitourinary vs breast | 2.38 | 1.33–4.24 | 0.003 |
| Thorax vs breast | 0.99 | 0.70–1.41 | 0.96 |
| Other sites vs breast | 2.26 | 1.47–3.47 | <0.001 |
| Age (per year) | 1.00 | 0.99–1.01 | 0.97 |
| Male sex | 0.72 | 0.44–1.17 | 0.19 |
| Treatment days | 1.05 | 1.02–1.08 | <0.001 |
| Palliative intent | 0.98 | 0.54–1.77 | 0.94 |
